# Trajectories of Alcohol Use Disorders and Their Differential Impact: A Population-Based Cohort Study in Goa, India

**DOI:** 10.1093/alcalc/agx038

**Published:** 2017-06-14

**Authors:** Abhijit Nadkarni, Helen A. Weiss, Bhargav Bhat, Vikram Patel

**Affiliations:** 1 Sangath, H No 451 (168), Bhatkar Waddo, Socorro, Porvorim, Bardez 403501, Goa, India; 2 London School of Hygiene and Tropical Medicine, Keppel Street, London, WC1E 7HT, UK; 3 Department of Global Health and Social Medicine, Harvard Medical School, 641 Huntington Avenue, Boston, MA 02115, USA

## Abstract

**Aims:**

The aim of this study was to examine trajectories of Alcohol Use Disorders (AUD) over a 6 year period and compare the bio-psycho-social correlates between these trajectories.

**Methods:**

Community-based cohort of 1899 adult men were interviewed in 2006–2008 and 2012–2014. AUD were assessed using the Alcohol Use Disorder Identification Test, and potential correlates including psycho-social problems, morbidity and physiological parameters were measured at follow-up. Logistic regression was conducted to estimate odds ratios (ORs) for the association of persistent and incident AUD, respectively, with the potential correlates. Analyses were weighted to account for sampling design, number of adults aged 18–49 years in the household and non-response.

**Results:**

Compared with men who had recovered from AUD, there was strong evidence (*P* < 0.001) that men with persistent AUD were more likely to have marital problems, tobacco use, and raised Gamma Glutamyl Transferase (GGT) and strong evidence (0.001 < *P* < 0.01) that they were more likely to have workplace problems, social problems, increased healthcare contact and raised Mean Corpuscular Volume (MCV). Compared with men who did not have AUD at baseline and follow-up, there was strong evidence (*P* < 0.001) that men with incident AUD were more likely to have workplace problems, social problems, marital problems, tobacco use, and raised GGT and strong evidence (0.001 < *P* < 0.01) that they were more likely to have hypertension, accident and injuries and Common Mental Disorders (CMD).

**Conclusion:**

This community-based longitudinal study of AUD, the first from a low and middle income country, clearly demonstrates significant health and social consequences of AUD in men and highlights the need for interventions for their treatment and prevention.

**Short Summary:**

Compared to persistent AUD, recovery from AUD has several benefits in health and social domains. Compared to developing new AUD, not having AUD has several benefits in health and social domains. Sustaining the state of not having AUD or recovery can lead to accumulation of health and social capital over time.

## BACKGROUND

An overwhelming majority of the estimated 2.9 million deaths globally due to substance use disorders are due to alcohol (Lim *et al.*, 2013). Alcohol Use Disorders (AUD) (hazardous drinking, harmful drinking and dependent drinking) account for about 10% of Disability Adjusted Life Years (DALYs) caused by mental and substance use disorders (Lim *et al.*, 2013).

Alcohol use behaviours change in frequency and intensity over time (Nadkarni *et al.*, 2016) and studies examining such behaviours at a single time point will not represent their course and progression. However almost all research examining the trajectories of alcohol use patterns has been conducted in developed countries and predominantly focuses on adolescent and young adult populations (Clark, 2004; Thatcher and Clark, 2006). The absence of evidence from Low and Middle Income Countries (LMIC) is a major knowledge gap. Evidence from developed countries is limited in its applicability to LMIC as AUDs, like many other health risk factors, are influenced temporally by a range of contextual factors such as socio-economic conditions, and access to health services. Hence, a better contextual understanding of the trajectory and correlates of AUDs will provide information relevant for clinical evaluation, prognostication and treatment; and will inform more appropriate policy and intervention development.

In India, almost half of all drinkers drink hazardously, with the pattern of hazardous drinking characterized by heavy drinking, daily or almost daily drinking, under-socialized, solitary drinking of mainly spirits and drinking to intoxication (Benegal, 2005). This results in high rates of alcohol-attributable mortality and prevalence of AUD relative to the per capita volume of alcohol consumed (Rehm *et al.*, 2009). Although there is substantial evidence from cross-sectional studies about the prevalence and correlates of AUD in India (Murthy *et al.*, 2010), the few existing cohort studies are limited by the short duration of follow-up, small sample sizes, clinic-based participants and limited data (Mohan *et al.*, 2002; Kar *et al.*, 2003; Kuruvilla and Jacob, 2007; Singh *et al.*, 2008).

This is the first study from a LMIC to follow a large community-based cohort and examine the longitudinal history of AUD. Our hypotheses were that men with persistent and incident AUD will have adverse bio-psycho-social correlates compared with those who have recovered from AUD or never had AUD, respectively.

## METHODS

### Setting

Goa, a state on the west coast of India, with a population of just over 1.4 million people (62% residing in urban areas) (Government of India, 2011). The epidemiological picture of drinking in Goa is characterized by a high proportion of AUD amongst male drinkers, and a range of adverse health and social impacts among those with AUD (Silva *et al.*, 2003; Gaunekar *et al.*, 2005; Costa *et al.*, 2007; Pillai *et al.*, 2013; Nadkarni *et al.*, 2016). The study was restricted to men because of the low prevalence of drinking in Indian women (Murthy *et al.*, 2010).

### Study design

From 2006 to 2008 a cross-sectional survey was conducted in the following socio-demographically heterogeneous study sites: urban (beach areas popular among tourists and a typical commercial and residential area) and rural areas (six contiguous villages) (Pillai *et al.*, 2013). The sampling frame was the electoral roll and the population-based sample was selected through a two-stage probability sampling procedure. From each randomly selected household a random male participant was selected from all eligible (18–49 years) men within that household. Refusal rates for randomly selected households were 1.5%. All men who were originally screened in the baseline survey were approached again to measure a range of potential correlates in 2012–2014.

### Baseline data

Baseline socio-demographic data (e.g. age, education) were collected. AUD was defined by the Alcohol Use Disorders Identification Test (AUDIT) using a cutoff score of ≥ 8 (Saunders *et al.*, 1993). The AUDIT has been validated and used in India (Pal *et al.*, 2004; Babu and Kar, 2010), and has been translated into Konkani (Goan vernacular), using a systematic translation-back translation method (Silva *et al.*, 2003).

### Follow-up procedures

At follow-up, all consenting participants were administered a self-report questionnaire and biological assessments by trained research workers using a standard protocol. The research workers were blind to the baseline AUD status of the participants and the study hypotheses to avoid non-random misclassification of potential correlates. Quality control was conducted by re-interviewing randomly selected participants (10%) by the research coordinator, direct observation of assessments done by the research workers and re-testing of randomly selected blood samples at an independent laboratory. If a participant was not available at the registered address, the research worker would visit again and the participant would be classified as a drop-out only after four failed visits. If the participant had moved house, then an attempt would be made to contact him over the phone and to obtain the new address from the neighbours. Finally, an attempt would be made to identify the participant's new address using the most recent electoral rolls.

### Follow-up data

Besides the AUDIT score, the following data was collected at assessment:

- Self-report using a structured questionnaire:
  1. Problems at work directly related to drinking: Questions which asked about any drinking related illness which kept the drinker from working on his regular activities for a week or more, losing or nearly losing a job because of drinking, people at work indicating that the drinker should cut down on drinking, and drinking hurting the chances for promotion/salary increases/bonuses/better jobs.
  2. Number of workdays lost due to poor health in past 28 days measured using an item derived from the WHO Health and Work Performance Questionnaire (HPQ) (Kessler *et al.*, 2003).
  3. Marital problems related to drinking: Questions which asked about a spouse getting angry about the drinking or the way the drinker behaved while drinking, and/or a spouse threatening to leave the drinker because of his drinking.
  4. Questions about physical abuse (slapped, hit, kicked, punched wife/partner or done something else that did or could have hurt her physically) of partner/spouse.
  5. Social problems: Questions used to assess social harm of drinking in the National Alcohol Surveys conducted by the Alcohol Research Group at Berkeley, USA and included those about getting into a heated argument while drinking, getting into a fight while drinking, prominent people from society (e.g. community elder) questioning or warning him because of his drinking, drinking contributing to him hurting or harassing someone else (emotionally, physically or sexually), getting into trouble because of drunk driving and being caught/ fined/threatened by the police or arrested for drunk driving (2001).
  6. Self-reported physical health problems (hypertension, head injury with loss of consciousness and diabetes) using questions from the 10/66 Dementia Research Group population-based research programme (Prince *et al.*, 2007).
  7. Accidents or injuries.
  8. Mental, Neurological and Substance Use (MNS) disorders
    - Current use of tobacco (smoked and/or chewed).
    - Current major depressive episode diagnosed using the Mini International Neuropsychiatric Interview (MINI 6.0) a validated short, structured diagnostic interview for DSM-IV and ICD-10 psychiatric disorders (Sheehan *et al.*, 1998), used in India (Salve *et al.*, 2012).
    - Common Mental Disorders (CMD) assessed using the validated 12 item General Health Questionnaire (GHQ 12) (Goldberg, 1978) which has been widely used in the study setting (Patel *et al.*, 2008).
  9. Health service utilization was measured using the adapted version of the validated Client Service Receipt Inventory (CSRI) (Chisholm *et al.*, 2000), which has been used in the study setting (Patel *et al.*, 2003).
- Clinical and biological parameters: blood pressure (BP), height, weight, Mean Corpuscular Volume (MCV) and Gamma Glutamyl Transferase (GGT). MCV value of > 92 fL and GGT value of > 50 IU/L were coded as abnormal. BMI of < 18.5 kg/m^2^ or > 24.9 kg/m^2^ was coded as ‘unhealthy BMI’.

### Ethics

Ethical approval was obtained from the Sangath Institutional Review Board (IRB), ethics committee of the London School of Hygiene and Tropical Medicine (LSHTM) and the Indian Council of Medical Research. Each research worker completed the NIH Protecting Human Research Participant online course. Results of the blood tests and their interpretation were fed back to the participants. Participants with abnormal health parameters were offered referral to the local primary healthcare centre. Participants diagnosed with AUD, major depressive episode or CMD were offered free clinical assessment and treatment with a psychiatrist.

### Statistical analyses

Inverse probability weights were applied to the data to account for the baseline sampling design, age distribution, rural and urban sample sizes, number of adults aged 18–49 years in the household (at baseline) and non-response (at baseline). The weights considered village or area population size, number of gender-specific adults aged 18–49 in the household, non-response, and a three category age distribution (18–29, 30–39, 40–49) within urban and rural areas. For example, for the village size factor, the sum of the weights for respondents in a village were adjusted such that the resulting sum of the weights for all individuals within the village divided by the total N (gender and urban/rural sample size) equalled the corresponding proportion created from the electoral rolls. Similarly, weights were normalized such that households where there were twice as many males aged 18–49 living in it compared to another household had twice the weighting factor. Baseline socio-demographic characteristics were compared by baseline AUD status using chi-squared or *t*-tests as appropriate. Baseline socio-demographic characteristics and AUD status were compared by follow-up status. Multivariable logistic regression was used to identify factors independently associated with loss to follow-up (LTFU).

A variable for drinking trajectories was created with the following categories: Persistent non-AUD (no AUD at baseline and follow-up), incident AUD (no AUD at baseline, AUD at follow-up), recovered AUD (AUD at baseline, no AUD at follow-up) and persistent AUD (AUD at baseline and follow-up). The baseline characteristics of men in these groups were compared using chi-squared test (for proportions), *t*-test (for means of two groups) and one way ANOVA (for means of more than two groups) as appropriate. Multivariable logistic regression was conducted to calculate ORs and 95% confidence intervals (CIs) for the association of persistent AUD (recovered AUD as comparator) and incident AUD (no AUD at baseline and follow-up as comparator) with bio-psycho-social correlates, adjusted for baseline socio-demographic factors. Sensitivity analyses were conducted using propensity scores. The Hosmer–Lemeshow test was applied to the logistic regression propensity model to establish goodness-of-fit to the data. The propensity score was then used to generate inverse probability of treatment (IPT) weights which were applied to the multiple regression models to account for selection bias resulting from missing data due to LTFU. All analyses were performed using STATA 14.

## RESULTS

At baseline the mean age of the cohort (*n* = 1899) was 32.8 (SD 8.6) years, 1077 (56.7%) resided in rural areas, 1077 (56.7%) were married or co-habiting, 93 (5.0%) were illiterate and 231 (12.2%) were unemployed. Compared with men without AUD, those with AUD were older (34.5 [7.8] years vs. 32.4 [8.8] years; *P* = 0.001), resided in urban areas (50.3% vs. 41.8%; *P* = 0.005), married (64.8% vs. 55.1%; *P* = 0.001), illiterate (6.8% vs. 4.6%; *P* = 0.10) and employed (90.7% vs. 87.2%; *P* = 0.07). On multivariable analysis the only variable strongly associated with AUD at baseline was urban residence (OR = 1.39; 95% CI 1.09–1.77; *P* < 0.001).

Over the follow-up period, 385 (20.3%) participants were LTFU (49 outward migration, and 336 refusals) and 62 (3.3%) had died. Having AUD at baseline was not associated with LTFU. On multivariable analysis the variables associated with LTFU were residence in urban areas (OR = 2.74; 95% CI 2.11–3.56; *P* < 0.001) and being literate (OR = 1.16; 95% CI 1.02–1.33; *P* < 0.02). Both these were included in the subsequent logistic regression models.

There were 1107 (76.3%; 95% CI 74.0–78.5) men who had no AUD at baseline and at follow-up, 106 (7.3%; 95% CI 6.0–8.8) had incident AUD, 117 (8.1%; 95% CI 6.7–9.6) had recovered AUD and 121 (8.3%; 95% CI 7.0–9.9) had persistent AUD. Men with persistent and recovered AUD tended to be older, living in urban areas, married and literate (Table 1).

**Table 1.** Comparison of baseline socio-demographic profile of the AUD trajectories

Variable	No AUD at baseline and follow-up, *n* (%)	Incident AUD, *n* (%)	Recovered AUD, *n* (%)	Persistent AUD, *n* (%)	*P* value
Total number	*N* = 1107 (76.3%)	*N* = 106 (7.3%)	*N* = 117 (8.1%)	*N* = 121 (8.3%)	
Mean age in years (SD)	32.6 (8.7)	32.6 (9.0)	34.0 (7.9)	34.7 (7.6)	0.03
Residence					
Rural	695 (77.7)	67 (7.5)	58 (6.5)	74 (8.3)	0.04
Urban	412 (74.0)	39 (7.0)	59 (10.6)	47 (8.4)	
Marital status					
Married or co-habiting	613 (73.7)	60 (7.2)	73 (8.8)	86 (10.3)	0.006
Never married or post-marital	494 (79.8)	46 (7.4)	44 (7.1)	35 (5.7)	
Educational status					
Illiterate	41 (64.1)	9 (14.1)	6 (9.4)	8 (12.5)	0.07
Literate	1034 (76.7)	93 (6.9)	109 (8.1)	113 (8.4)	
Employment status					
Unemployed	132 (79.0)	12 (7.2)	11 (6.6)	12 (7.2)	0.80
Employed	975 (75.9)	94 (7.3)	106 (8.3)	109 (8.5)	

Table 2 compares prevalence of each correlate by AUD trajectory and the association between AUD trajectory and correlates. The prevalence of the following correlates was highest among men with persistent AUD, followed by those with incident and then recovered AUD: workplace problems, social problems, marital problems, tobacco use, CMD, healthcare contact and raised GGT. Conversely, the prevalence of hypertension was highest for men with recovered AUD, followed by those with incident and persistent AUD. The prevalence of raised MCV was highest for men with persistent AUD, followed by those with incident and persistent AUD.

**Table 2.** Outcomes associated with AUD trajectories

Variable	Recovered AUD, *n* (%)^a^	Persistent AUD, *n* (%)^a^	No AUD at baseline and follow-up, *n* (%)^a^	Incident AUD, *n* (%)^a^	*P* value	Multivariable analysis^b^	
						Persistent AUD [Comparator is recovered AUD] OR (95% CI)	Incident AUD [Comparator is no AUD at baseline and follow-up] OR (95% CI)
Total number	*N* = 117	*N* = 121	*N* = 1107	*N* = 106			
Social problems							
Workplace problems since baseline interview	3 (4.7)	32 (30.8)	7 (2.4)	24 (25.0)	<0.001	9.1 (2.5–33.3)	12.2 (4.8–31.0)
Marital problems since baseline interview	4 (6.3)	50 (48.5)	19 (7.2)	36 (40.0)	<0.001	12.9 (4.2–39.0)	8.1 (4.1–16.0)
Social problems since baseline interview	1 (1.5)	29 (26.9)	6 (2.0)	18 (17.8)	<0.001	19.7 (2.6–151.6)	10.1 (3.7–27.5)
Lost ≥ 1 workdays due to poor health in past 28 days	14 (13.7)	20 (22.5)	127 (12.6)	15 (16.3)	0.27	1.6 (0.7–3.5)	1.5 (0.8–2.7)
Physical abuse of partner/spouse in past 12 months	4 (4.0)	11 (11.6)	24 (2.8)	6 (7.2)	0.14	2.9 (0.8–10.1)	2.4 (0.9–6.1)
Physical health problems							
Hypertension diagnosed after baseline interview	18 (15.4)	27 (22.3)	123 (11.1)	22 (21.2)	0.36	1.6 (0.8–3.3)	2.5 (1.5–4.4)
Head injury with loss of consciousness after baseline interview	9 (7.7)	13 (10.9)	61 (5.5)	8 (7.6)	0.60	1.3 (0.5–3.4)	1.4 (0.6–3.1)
Diabetes diagnosed after baseline interview	8 (6.8)	20 (16.7)	71 (6.4)	11 (10.6)	0.06	2.8 (1.1–7.0)	2.2 (1.1–4.5)
Accidents or injuries in past 12 months	16 (13.7)	30 (25.0)	113 (10.2)	18 (17.1)	0.07	1.8 (0.9–3.6)	1.9 (1.1–3.3)
Mental health and substance use/abuse							
Used tobacco in past 12 months	53 (45.3)	91 (75.2)	266 (24.0)	63 (59.4)	<0.001	3.5 (1.9–6.4)	4.5 (2.9–7.2)
Current major depressive episode	3 (2.6)	10 (8.4)	32 (2.9)	3 (2.9)	0.06	3.5 (0.9–13.5)	1.0 (0.3–3.3)
Common mental disorders	2 (1.7)	11 (9.2)	17 (1.5)	7 (6.7)	0.04	4.8 (0.9–24.6)	3.6 (1.4–9.2)
Health service utilization							
Contact with health worker in past 2 months	33 (28.2)	55 (45.5)	422 (38.1)	46 (43.8)	0.01	2.1 (1.2–3.7)	1.3 (0.9–2.0)
Admitted to hospital in the past two months	2 (1.7)	8 (6.6)	21 (1.9)	4 (3.8)	0.16	4.1 (0.8–21.4)	1.9 (0.6–5.9)
Biological parameters							
Hypertension	16 (14.2)	5 (4.3)	78 (7.1)	11 (10.7)	0.04	0.3 (0.1–0.8)	1.6 (0.8–3.3)
Unhealthy BMI	48 (43.6)	58 (51.8)	469 (43.2)	46 (44.7)	0.42	1.3 (0.8–2.3)	1.1 (0.8–1.7)
Raised MCV	34 (40.5)	70 (63.6)	245 (27.1)	34 (36.6)	<0.001	2.5 (1.4–4.7)	1.5 (1.0–2.4)
Raised GGT	21 (25.0)	58 (52.7)	83 (9.1)	33 (35.1)	<0.001	3.4 (1.8–6.5)	6.2 (3.7–10.3)

^a^Denominator is the number of participants in the trajectory with follow-up data for the relevant outcome available.

^b^Adjusted for baseline age, education, employment status, marital status, area of residence, socio-economic status.

### Multivariable analysis

Compared with men who had recovered from their AUD, there was very strong evidence (*P* < 0.001) that men with persistent AUD were more likely to have marital problems (OR 12.9; 95% CI 4.2–39.0), tobacco use (OR 3.5; 95% CI 1.9–6.4), and raised GGT (OR 3.4; 95% CI 1.8–6.5) and strong evidence (0.001 < *P* < 0.01) that they were more likely to have workplace problems (OR 9.1; 95% CI 2.5–33.3), social problems (OR 19.7; 95% 2.6–151.6), increased healthcare contact (OR 2.1; 95% CI 1.2–3.7) and raised MCV (OR 2.5; 95% CI 1.4–4.7). In addition, there was evidence (0.01 < *P* < 0.05) that men with persistent AUD were more likely to have diabetes (OR 2.8; 95% CI 1.1–7.0), and less likely to have hypertension (OR 0.3; 95% CI 0.1–0.8). Compared with men who did not have AUD at baseline and follow-up, there was very strong evidence (*P* < 0.001) that men with incident AUD were more likely to have workplace problems (OR 12.2; 95% CI 4.8–31.0), social problems (OR 10.1; 95% CI 3.7–27.5), marital problems (OR 8.1; 95% CI 4.1–16.0), tobacco use (OR 4.5; 95% CI 2.9–7.2), and raised GGT (OR 6.2; 95% CI 3.7–10.3) and strong evidence (0.001 < *P* < 0.01) that they were more likely to have hypertension (OR 2.5; 95% CI 1.5–4.4), accident and injuries (OR 1.9; 95% 1.1–3.3) and CMD (OR 3.6; 95% CI 1.4–9.2). In addition, there was evidence that men with incident AUD were more likely (0.01 < *P* < 0.05) to have diabetes (OR 2.2; 95% CI 1.1–4.5). Finally, there were some associations with persistent AUD which had large OR (>4.0), but wide confidence intervals including 1 and these included CMD and admission to hospital (Table 2). The results remained similar even after adjusting for the propensity scores.

## DISCUSSION

This study describes the different longitudinal trajectories of AUD and the correlates associated with these trajectories in a community cohort of men from India. There was no clear and consistent differential pattern of association of various socio-demographic correlates with AUD trajectories. However, there was a clear pattern of differential association of various correlates with AUD trajectories. Persistent AUD and incident AUD were associated with a wide range of adverse correlates compared to recovered AUD and those with no AUD at baseline and follow-up, respectively. Hence, while interpreting our findings we need to consider the coherence of the narrative resulting from the pattern of the highly statistically significant and relatively less statistically significant associations. Furthermore, we also need to take into consideration the wide 95% confidence intervals of some of the statistically significant findings, as they reflect limited precision of those findings.

We found that age, area of residence and marital status were predictors of drinking trajectories. However, the clinical utility of such socio-demographic predictors is doubtful as the same socio-demographic factor might predict more than one trajectory, e.g. illiteracy predicted development of new AUD, recovery and persistence of AUD. Some studies have been able to identify similar long-term predictors of drinking outcomes. Generally older people were seen to have poorer outcomes and particularly in less severe types of AUD it was found that the younger the subject, higher the likelihood of improvement (Ojesjo, 1981). Other factors that have been found to predict prognosis in AUD include severity of AUD, financial condition, health status, interpersonal relations, etc.; and a favourable prognosis predicted by emotional and social stability, and a satisfactory combination of work and interpersonal adaptation (Rosenberg, 1993; Sobell *et al.*, 2000). However overall the evidence on predictors of outcome is not substantial (Gual *et al.*, 1999) and most that exists is in clinical samples. It is difficult to compare population studies like ours with clinical samples, as there are important differences in sampling strategies, nature of the sample, setting, etc.

Our findings of correlates of AUD trajectories are consistent with evidence about outcomes of drinking trajectories from developed countries which indicates that individuals with AUD experience more problems during the follow-up interval, than individuals who become controlled drinkers or abstainers (Vaillant and Milofsky, 1982; McCabe, 1986; Finney and Moos, 1991). These include social problems, accidents and injuries, psycho-social problems, higher rates of health problems and hospitalizations in sustained AUD compared to recovered AUD (Ojesjo, 1981; McCabe, 1986; Gual *et al.*, 2009). More importantly, our key findings are that recovered AUD and not having AUD at baseline and follow-up has better bio-psycho-social correlates compared to persistent AUD or incident AUD, respectively. This is consistent with other studies that have highlighted the resources that can be accumulated over time (e.g. health, employment, strong social relations) as the state of not having an AUD is sustained (Levy *et al.*, 1981; Brown and Schuckit, 1988; Brown *et al.*, 1991; Maisto *et al.*, 1998; Dennis *et al.*, 2007). Such positive changes in a range of outcomes (including drinking outcomes) form an important component of ‘recovery’ viz a multidimensional construct including not having AUD, as well as improvements in other domains (e.g. mental or physical), and satisfaction with environment and relationships with others (Garner *et al.*, 2014).

One of the major strengths of this study is that it is a community sample. Most studies examining the progression and correlates in AUD have drawn samples from clinic populations that are biased by the non-inclusion of less severe forms of AUD and also by other confounders like treatment provision. Such studies are not very useful in understanding the progression and correlates of AUDs in resource poor settings. This is the first of its kind study from India or indeed any LMIC. It allows us to understand the differential association of correlates based on AUD trajectories in the context of a LMIC. The large size of the sample, reasonably long period of follow-up, good follow-up rates and use of structured questionnaire are other strengths of the study. Our study has some limitations as well. We used the electoral register as a sampling frame as it is the most complete and accessible national source of residential addresses. However, this strategy can lead to selection bias for several reasons such as people not choosing to register themselves, and recent migrants not being represented on the electoral rolls. Some of the participants in the cohort might have received treatment for their AUD which might have influenced the course as well as the impact of the various trajectories. However, considering the scarcity of services for AUD in the study setting it is unlikely that many participants would have received such treatment. Although we have defined the trajectory based on the presence/absence of AUD at two time points, due to the fluctuating course of AUD, it is possible that someone who we have labelled as persistent AUD has actually had a period of abstinence in the interim period. Furthermore, since we did not have baseline data of the various correlates measured at follow-up, we could not adjust for those at baseline. This in turn means that we can only make conclusions regarding associations between the AUD trajectories and adverse correlates, but cannot make definitive conclusions about direction of causality, e.g. workplace problems. However, for some of the associations reverse causality does not appear plausible and it is possible to make assumptions about causality. For example, although it is possible that social or interpersonal problems could be due to drinking or lead to drinking, someone starting to drink because they have raised GGT appears implausible. Finally, many measurements in our study, including alcohol use, are self-reported and could be biased due to socially desirable response.

Our findings have clinical, research and policy implications. The key message is that recovery from AUD holds several benefits over persistent AUD. Furthermore, developing new AUD is associated with a range of adverse bio-psycho-social correlates. Hence, in LMIC, it is important for policy makers to invest in the delivery of interventions that are focused on prevention and management of AUD, a set of disorders which have one of the largest treatment gaps amongst all mental, neurological and substance use disorders (Kohn *et al.*, 2004). Despite the differences in our setting from previous studies from developed countries, our findings show the universality of the adverse correlates associated with the various AUD trajectories. This could mean that the general principles underlying policies, services and interventions from developed countries could be adopted in LMIC after appropriate contextual adaptations. We had low power to detect some of the associations that we have described above (such as physical abuse) and these need to be examined in larger studies powered to test these associations as these are critical issues deserving further exploration. Finally, a key research question that needs to be examined in greater detail is the predictors of the various AUD trajectories as these will inform the development of appropriate programmes for the prevention and treatment of AUD.
